# Supplementary material for: Quantum confined peptide assemblies with tunable visible to near-infrared spectral range
Source: Nat Commun. 2018 Aug 13;9:3217. doi: 10.1038/s41467-018-05568-9 (PMC6089888; doi:10.1038/s41467-018-05568-9)
Supplement: Supplementary file 1 — Supplementary Information [file 41467_2018_5568_MOESM1_ESM.pdf]

Supplementary Information for

Quantum Confined Peptide Assemblies with Tunable Visible to Near-Infrared Spectral Range

Kai Tao, Zhen Fan, Leming Sun, Pandeeswar Makam, Zhen Tian, Mark Ruegsegger, Shira Shaham-Niv, Derek Hansford, Ruth Aizen, Zui Pan, Scott Galster, Jianjie Ma, Fan Yuan, Mingsu Si, Songnan Qu, Mingjun Zhang, Ehud Gazit, Junbai Li

Supplementary Figures

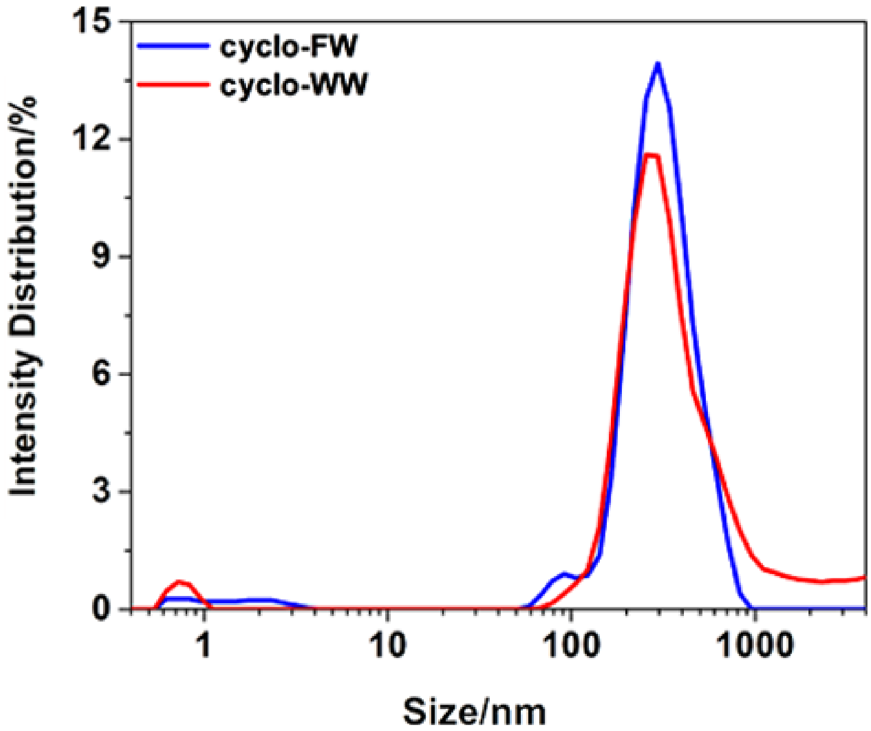

Supplementary Figure 1 DLS characterization of cyclo-dipeptides self-assembly (5.0 mM in MeOH). The results demonstrate that both cyclo-dipeptides self-assembled into larger particles several hundred nanometers in size.

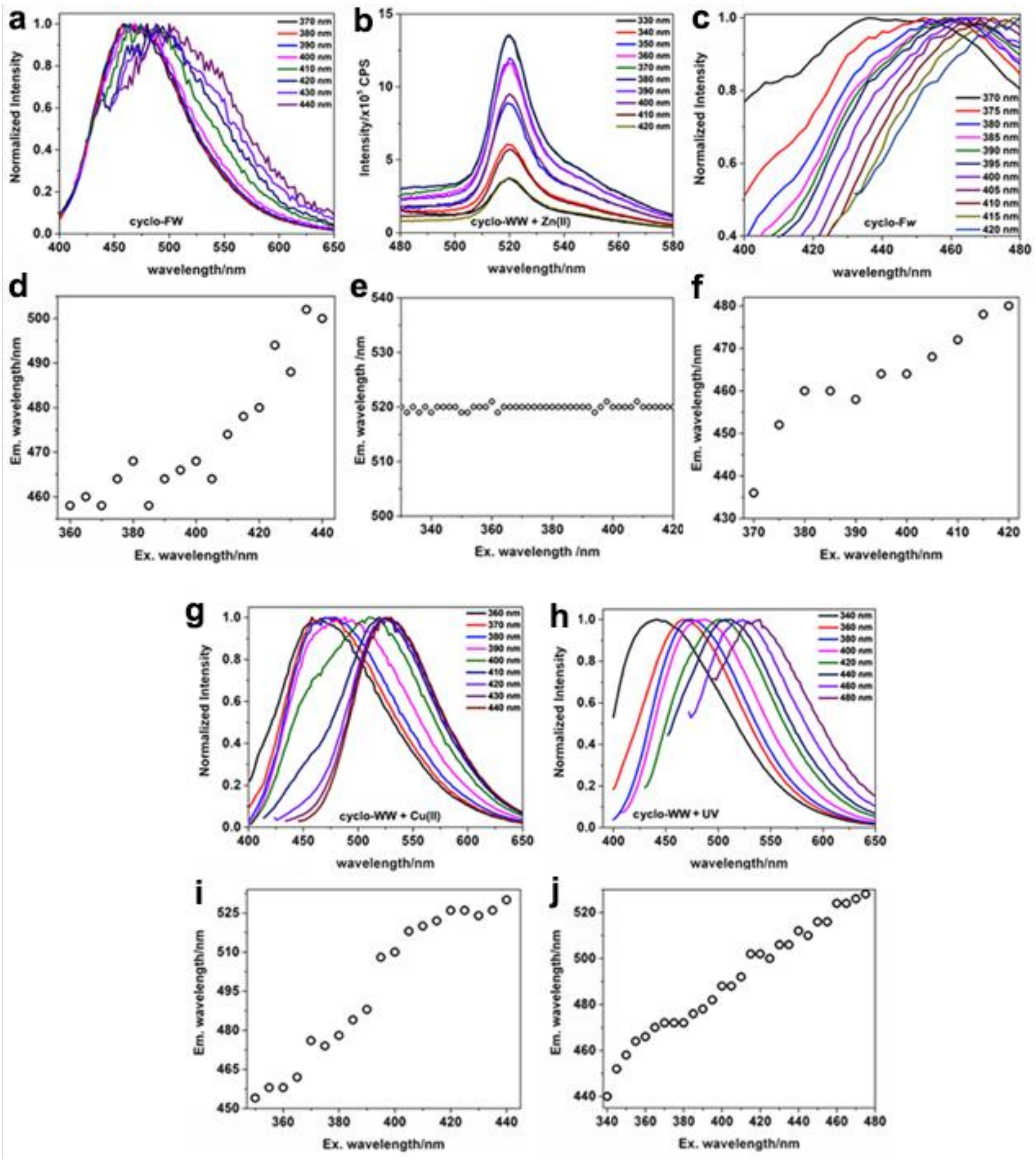

Supplementary Figure 2 Fluorescent emissions of cyclo-dipeptides self-assemblies in MeOH. (a-c, g, h) Fluorescent emission spectra and **(d-f, i, j)** wavelength evolution versus excitation of cyclo-FW, cyclo-WW+Zn(II), cyclo-F*w*, cyclo-WW+Cu(II) and cyclo-WW+UV, respectively. The maximal emission of cyclo-WW+Zn(II) (**b, e**) kept constant regardless of the excitation wavelength. In contrast, in all other cases, the maximal emission red shifted with excitation.

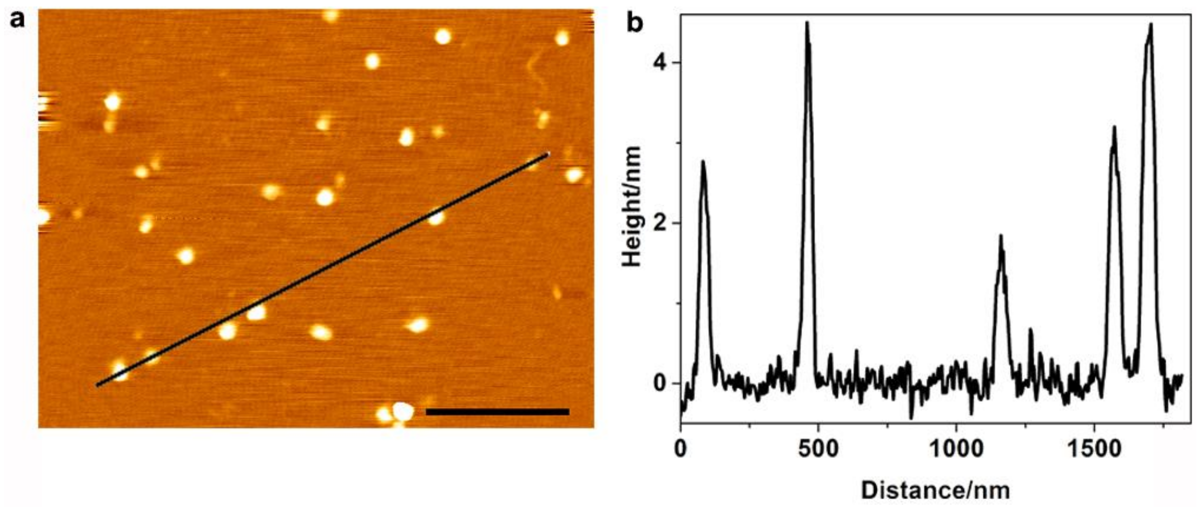

Supplementary Figure 3 Height measurement of dimers self-assembled by cyclo-WW+Zn(II) in MeOH. a, AFM micrograph of the dimers. Scale bar: 500 nm. **b,** The cross-section profile corresponding to the black line in (**a**), demonstrating that the size of the dimers was approximately 3.0 nm.

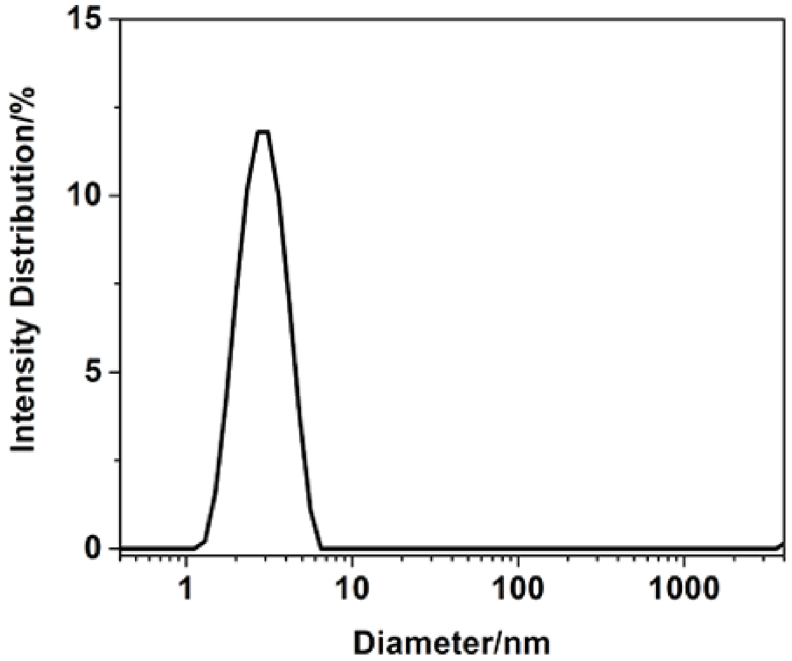

Supplementary Figure 4 DLS analysis of cyclo-WW+Zn(II) in MeOH. The single peak at 2.88 nm demonstrates that only dimers were present in the solution.

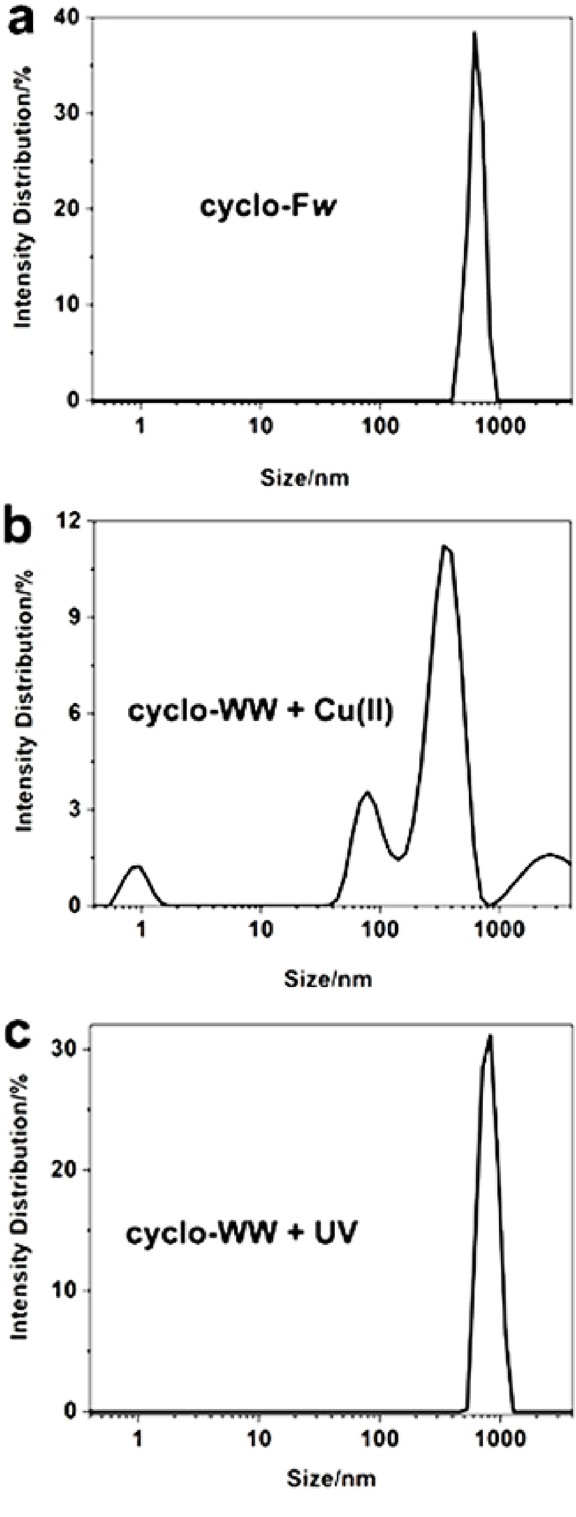

Supplementary Figure 5 DLS characterization of the size distributions of cyclo-dipeptide self-assemblies in MeOH. a, cyclo-Fw. **b,** cyclo-WW+Cu(II). **c,** cyclo-WW+UV. The results demonstrate that in all three samples, the cyclo-dipeptides self-assembled into larger supramolecular structures, several hundred nanometers in size.

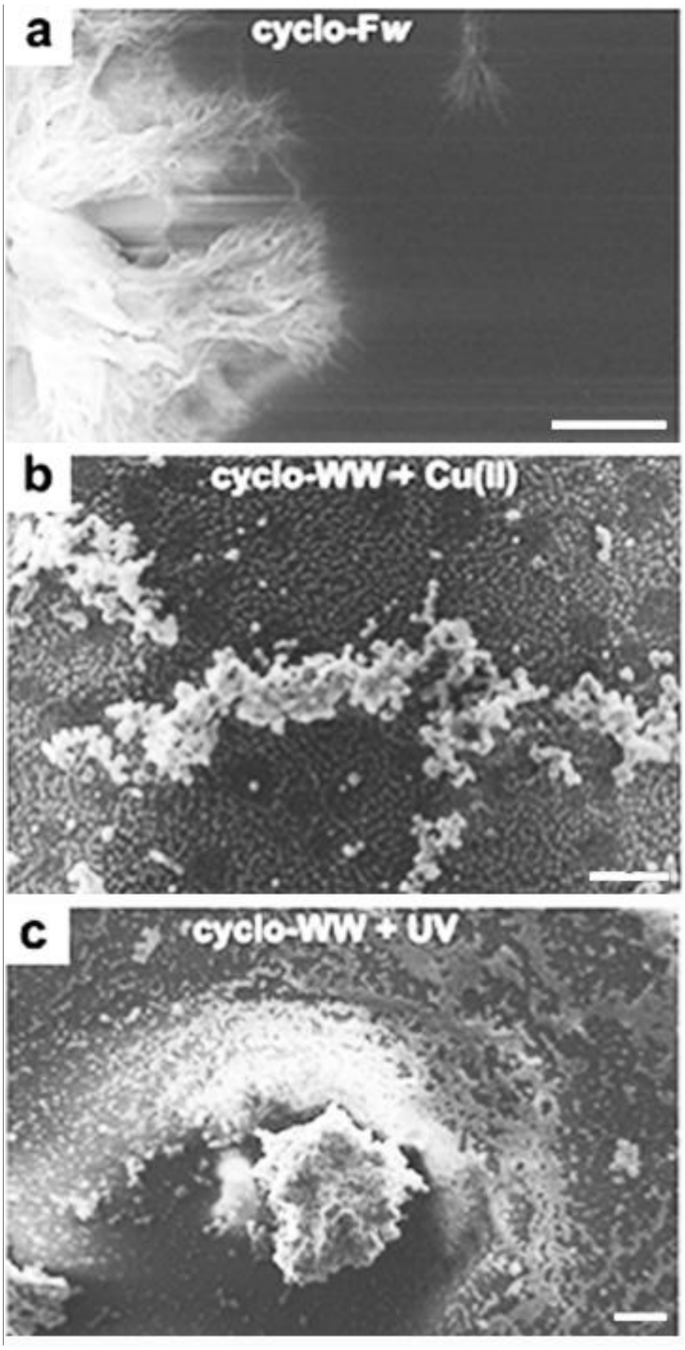

Supplementary Figure 6 Additional SEM images showing the aggregation of cyclo-dipeptides self-assemblies. **a**, The nano-flower structures comprised of intertwined nanofibrils self-assembled by cyclo-F*w*. Scale bar: 1 μm. **b**-**c**, Spherical nanoparticle aggregations by (**b**) cyclo-WW+Cu(II) and (**c**) cyclo-WW+UV. Scale bar: 10 μm.

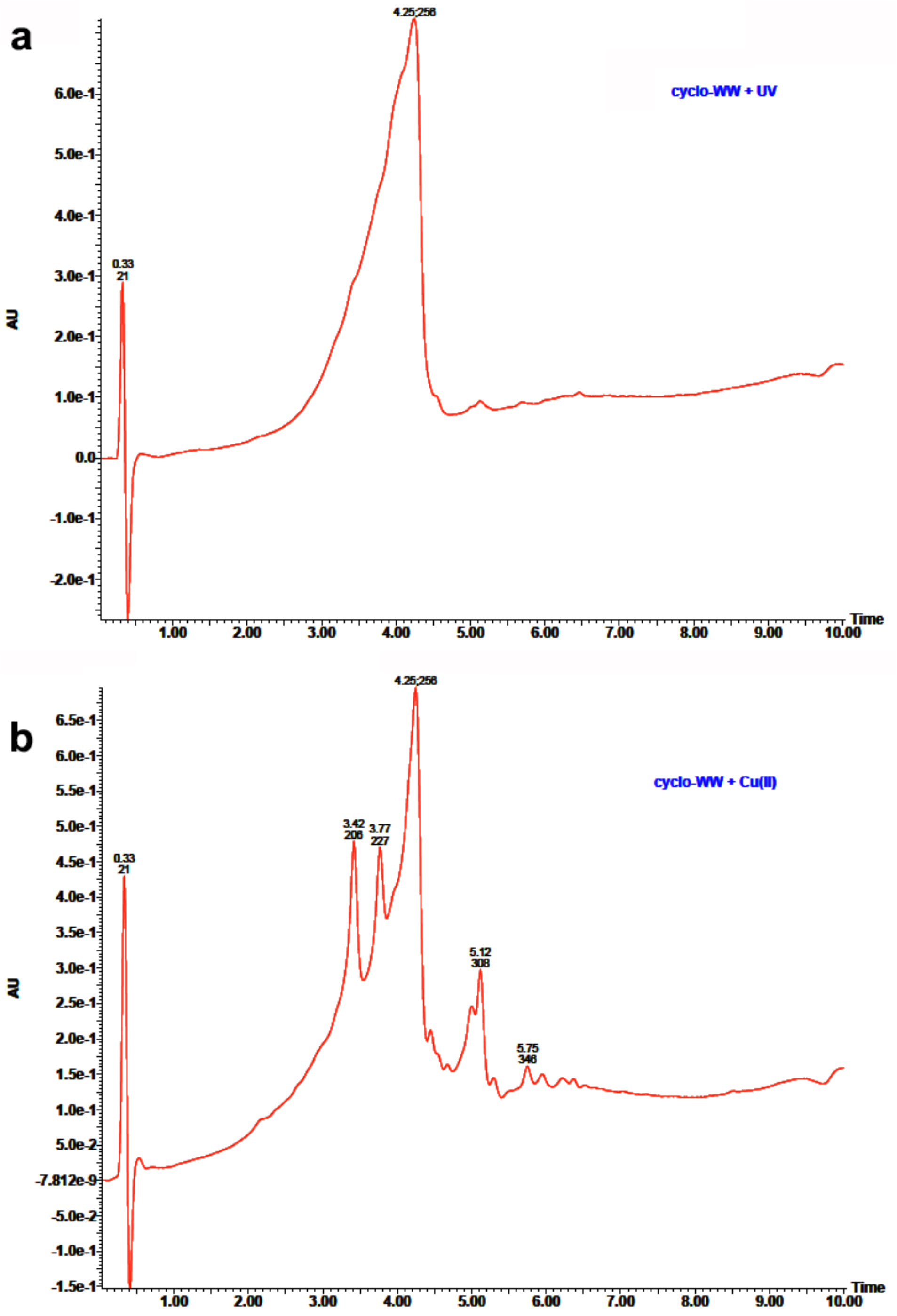

Supplementary Figure 7 HPLC profiles of the oxidized cyclo-WW. a, cyclo-WW under UV illumination (365 nm) for one week. **b,** cyclo-WW under Cu(II) oxidation for one month. The results demonstrate that the oxidized cyclo-WW was very pure after UV illumination, showing only one dominant peak, while several accompanying peaks existed in the cyclo-WW+Cu(II) system due to redox reactions.

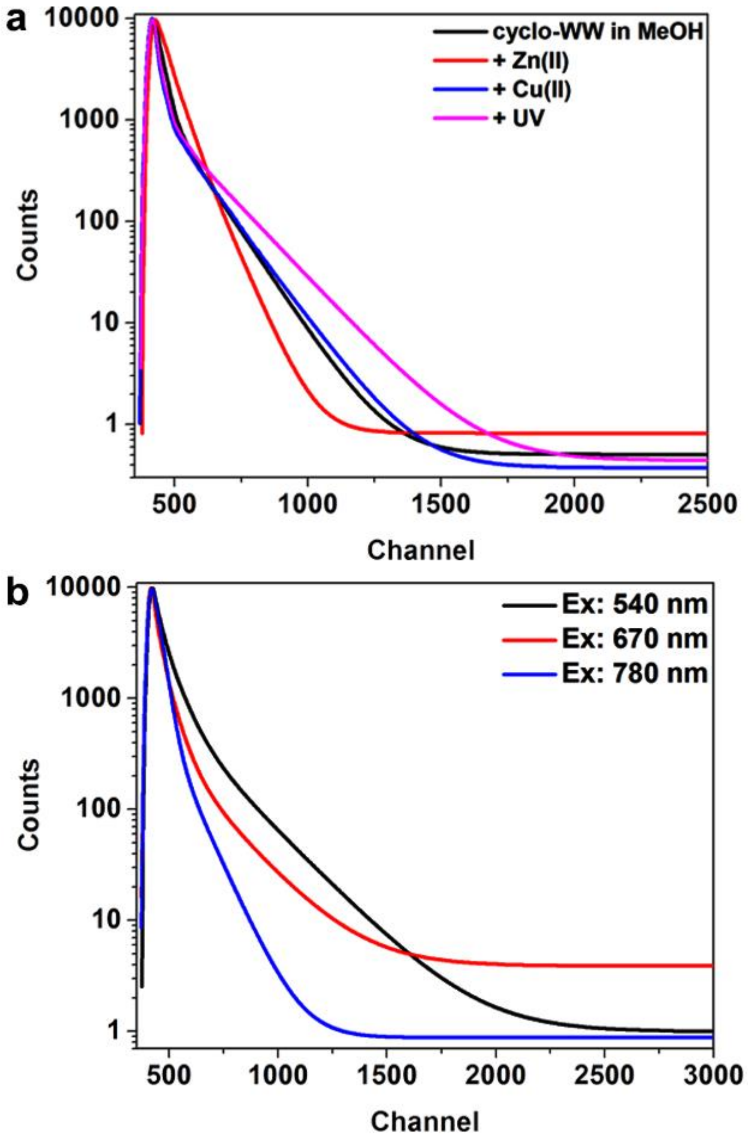

Supplementary Figure 8 Fluorescent decay curves of cyclo-WW self-assemblies. a, cyclo-WW in MeOH (cyclo-WW: 370/425; + Zn(II): 370/520; + Cu(II)--370: 370/465; + UV: 370/465). **b,** cyclo-WW+Zn(II) in DMSO (540/610; 670/712; 740/817). The fitted fluorescent lifetime is summarized in Fig. 2f in the main text.

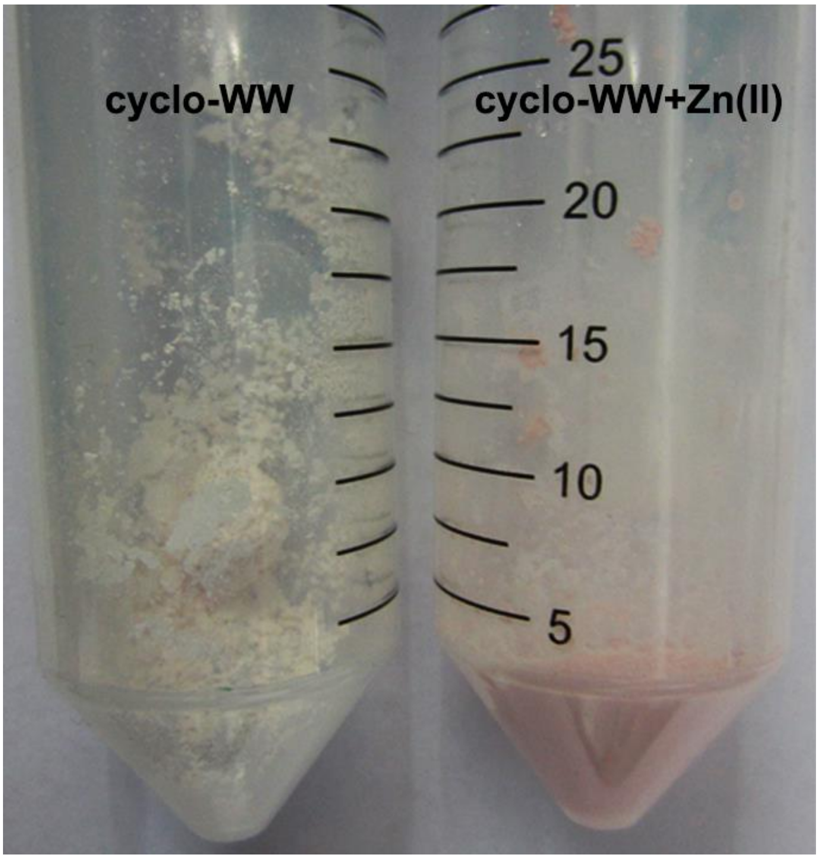

Supplementary Figure 9 Photographic presentation of the color change of dried cyclo-WW before and after coordination with Zn(II). Left: cyclo-WW assemblies after MeOH evaporation; Right: cyclo-WW+Zn(II) after MeOH evaporation. After complexation with Zn(II), the color changed from the original white/light yellow into pink.

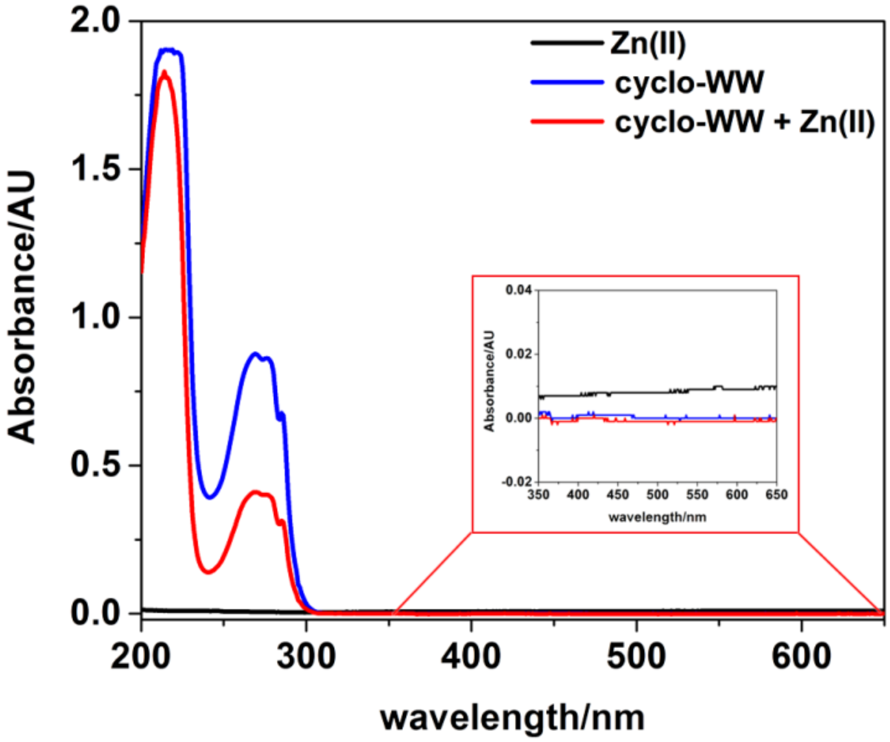

Supplementary Figure 10 UV-Vis absorption spectra of cyclo-WW monomers (0.05 mM in MeOH) in the absence or presence of Zn(II). The inset shows the amplified profile between 350 nm and 650 nm. The results demonstrate that no new peak appeared when introducing Zn(II) into a cyclo-WW monomer solution, indicating that the cyclo-WW monomer did not complex with Zn(II).

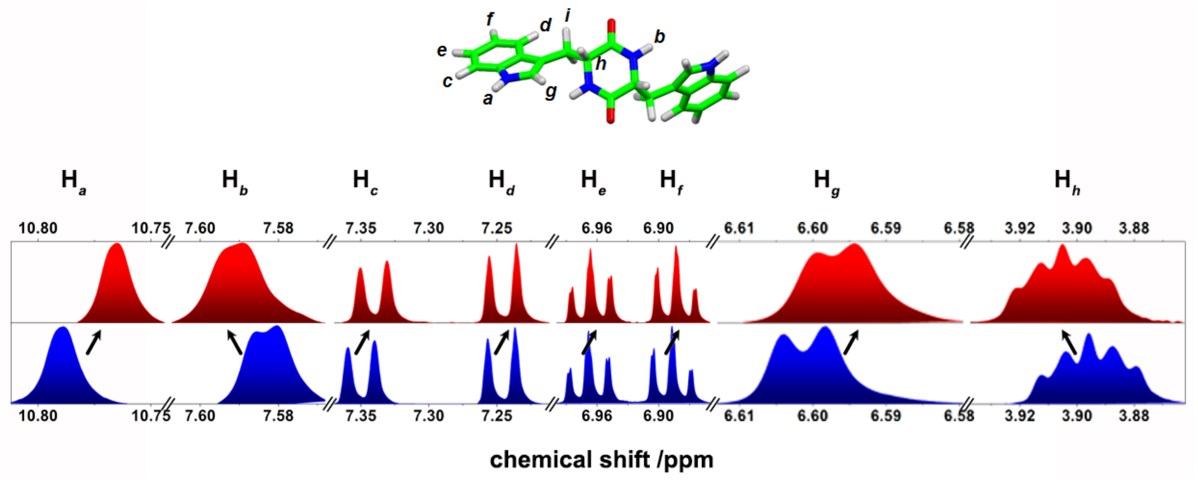

Supplementary Figure 11 ^{1}**H-NMR spectra of cyclo-WW in the absence (blue) or presence (red) of Zn(II).** The hydrogen atoms in different chemical environments were marked with italicized alphabet letters. The results demonstrate that the hydrogen atoms in backbone diketopiperazine ring showed downfield shifts, while the hydrogen atoms in side-chain aromatic indole rings presented upfield shifts.

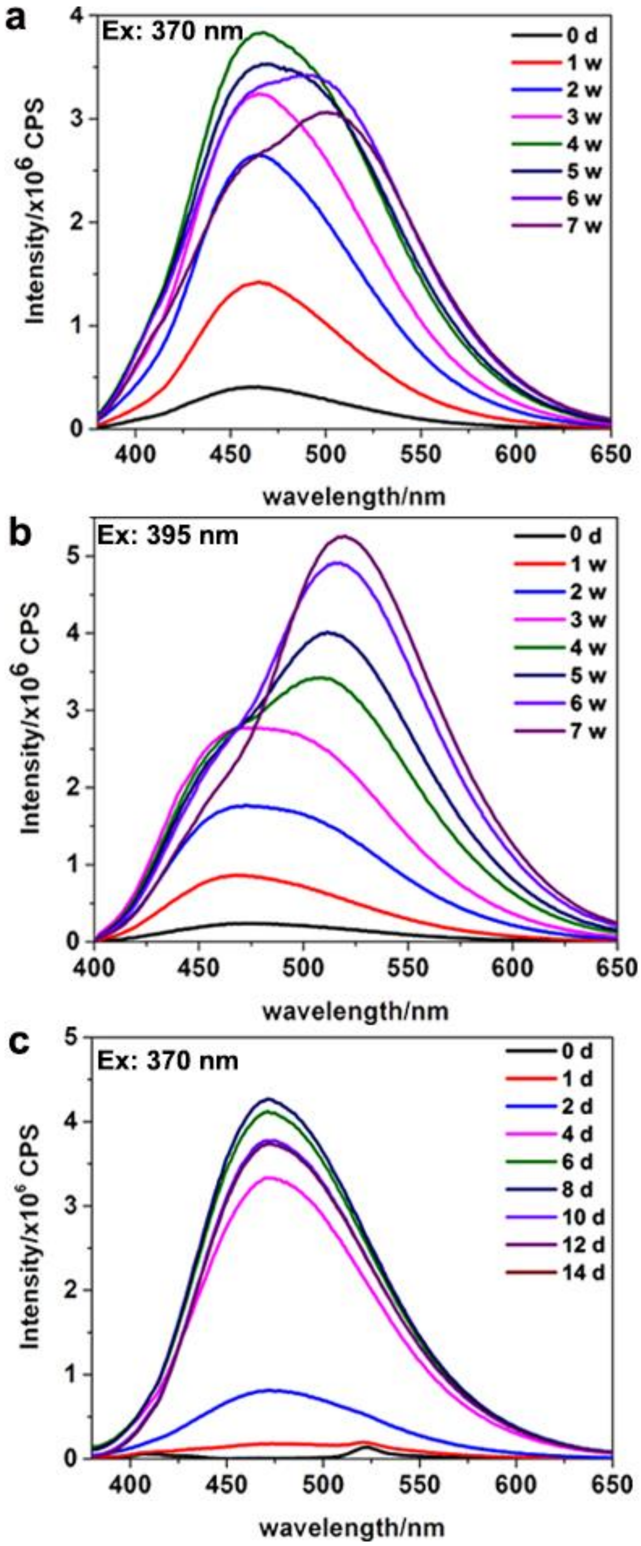

Supplementary Figure 12 Time-resolved fluorescent emission spectra of cyclo-dipeptides self-assemblies. a, cyclo-WW+Cu(II) excited at 370 nm. **b,** cyclo-WW+Cu(II) excited at 395 nm. **c,** cyclo-WW+UV excited at 370 nm. The extracted maximal emissions plotted against time are shown in Supplementary Fig. 13. (d: day; w: week.).

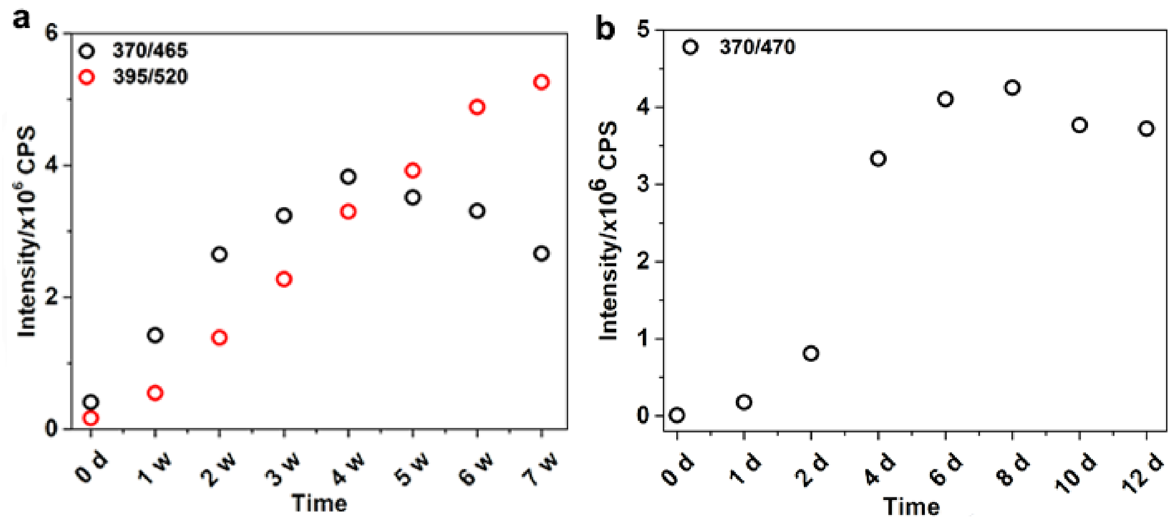

Supplementary Figure 13 Time-resolved fluorescent emission extracted from the fluorescence spectra presented in Supplementary Fig. 12. a, cyclo-WW+Cu(II). **b,** cyclo-WW+UV.

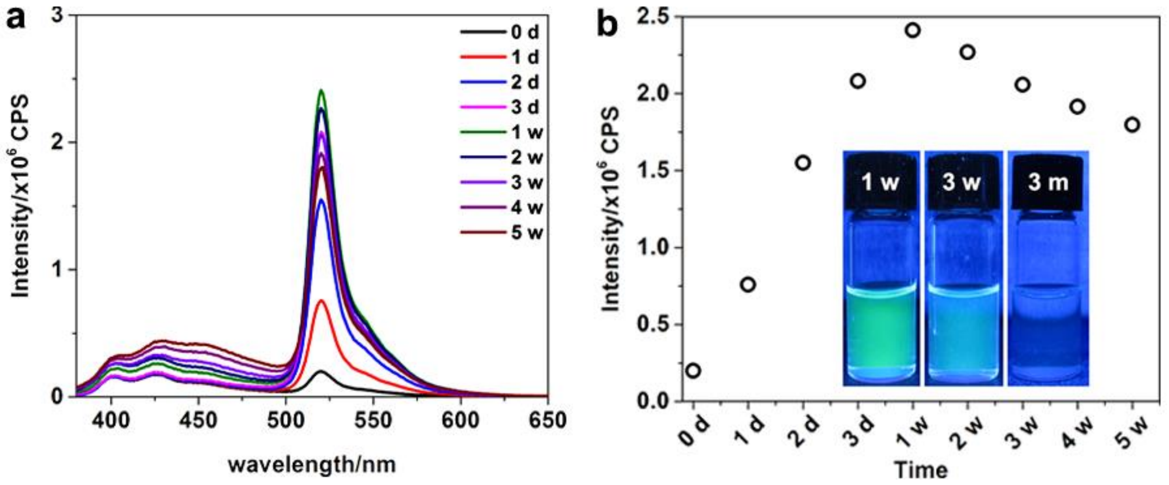

Supplementary Figure 14 Time-resolved fluorescence emission of cyclo-WW+Zn(II) MeOH solution. a, Fluorescent emission spectra. **b,** Plot of extracted emission at 520 nm *vs.* time. The inset in (**b**) shows the fluorescent color evolution over time (d: day; w: week, m: month).

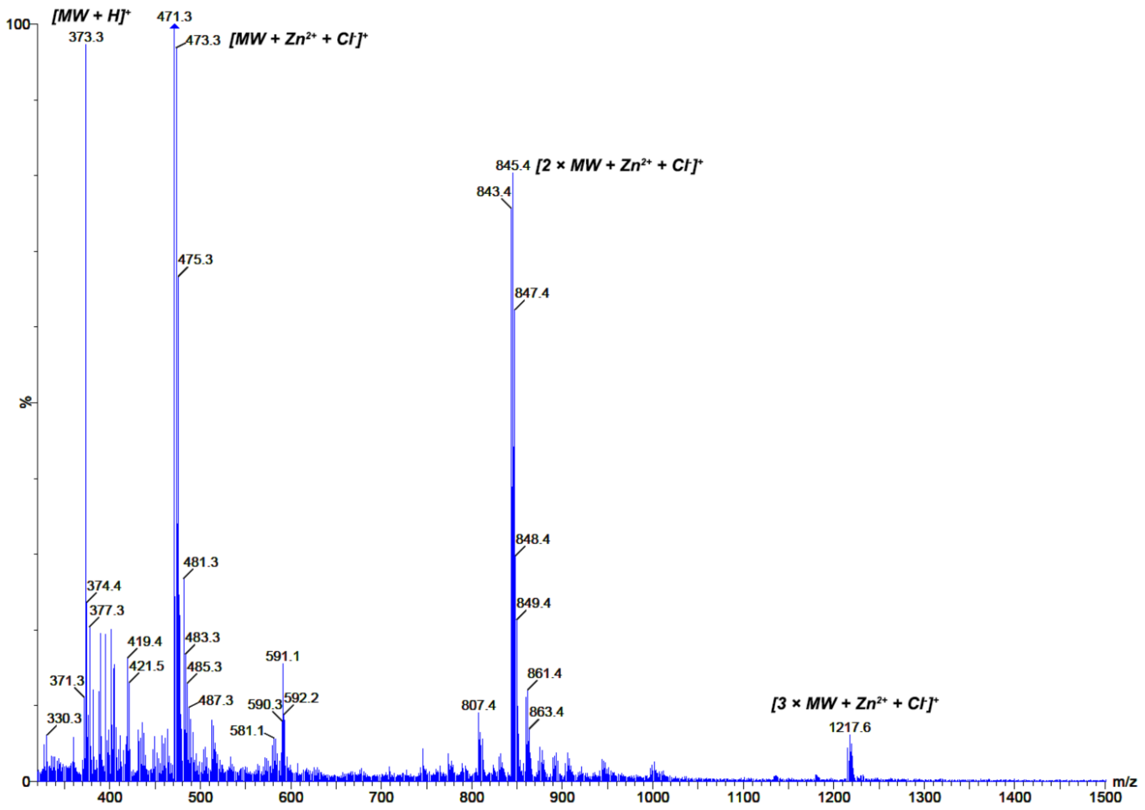

Supplementary Figure 15 MS spectrum of cyclo-WW+Zn(II) in MeOH. Since the zinc ion has two positive charges, a chloride negative ion was always accompanied, ensuring the complex ion has only one positive charge.

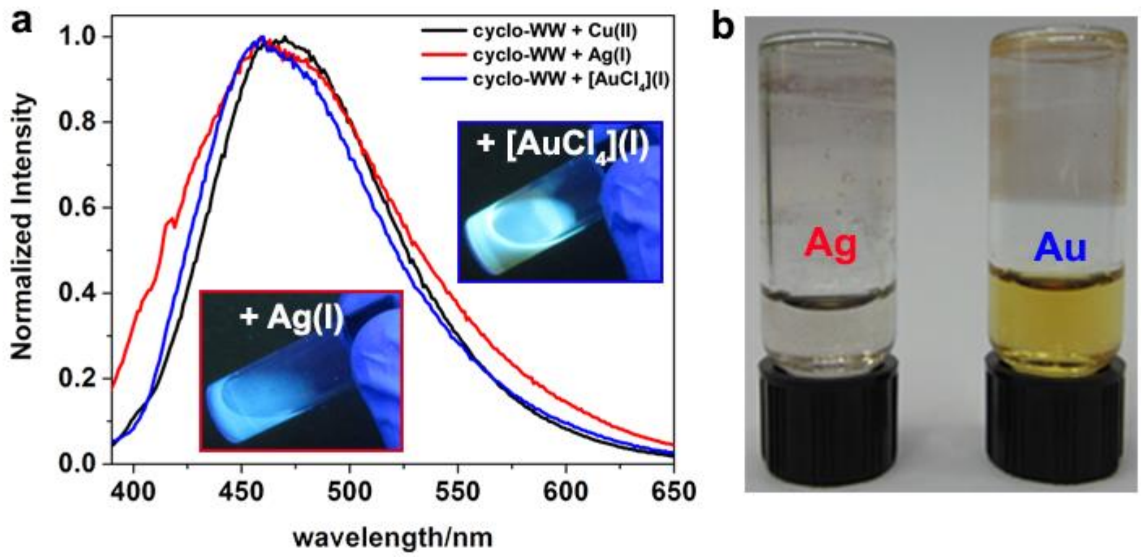

Supplementary Figure 16 Photoluminescent properties of cyclo-WW+Ag(I) and cyclo-WW+[AuCl$_4$](-I). a, Fluorescent emission spectra. The insets show the fluorescent color of the sample solutions under UV light (365 nm). The results revealed that cyclo-WW+Ag(I) and cyclo-WW+[AuCl$_4$](-I) had the same emissions as cyclo-WW+Cu(II). **b,** Photos demonstrating the reduced Ag and Au metals adsorbed on the vial walls.

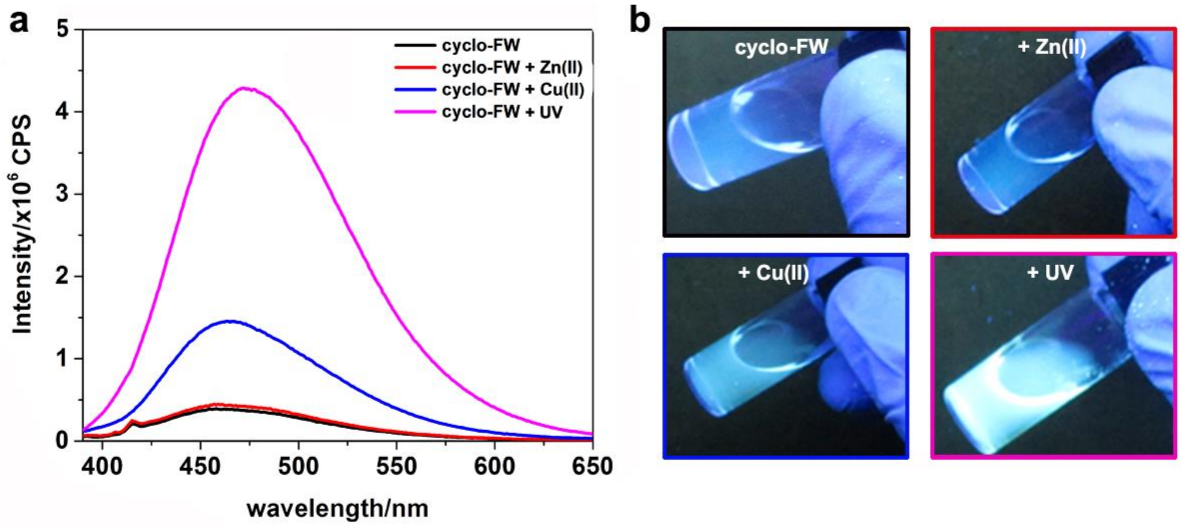

Supplementary Figure 17 Fluorescent spectra of cyclo-FW self-assemblies in the presence of metal ions or UV irradiation. a, Fluorescent emission spectra of cyclo-FW in the absence or presence of Zn(II), Cu(II) and UV irradiation. **b,** Photos showing the color of the solutions under UV light (365 nm). The results revealed similar fluorescent emission in the absence or presence of Zn(II), indicating that cyclo-FW did not coordinate with Zn(II). In contrast, in the presence of Cu(II) and UV irradiation, intense fluorescence was detected, demonstrating the different mechanisms of modulating the bioinspired supramolecular fluorescence by Zn(II) *vs*. Cu(II)/UV.

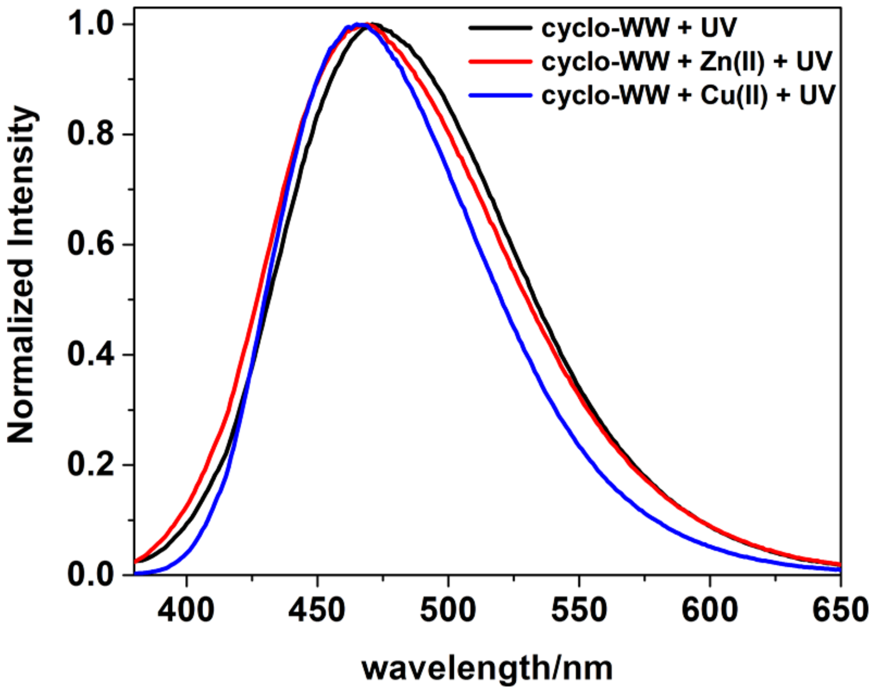

Supplementary Figure 18 Fluorescent spectra of UV-irradiated (365 nm) cyclo-WW in the absence or presence of metal ions. The similar emission spectra indicated that the oxidized cyclo-WW could not complex with metal ions.

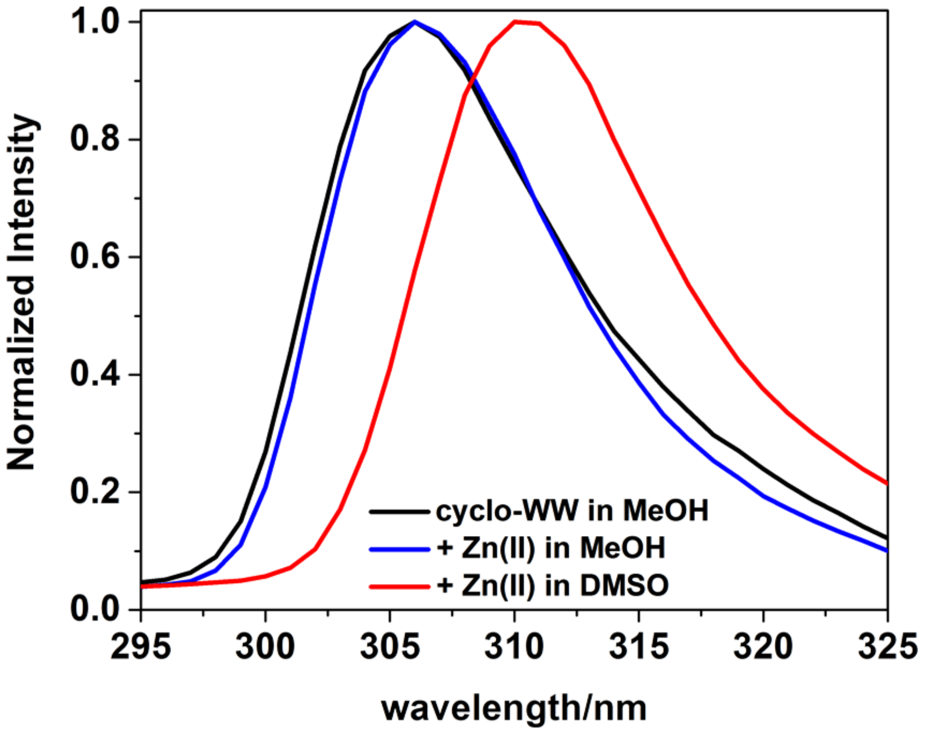

Supplementary Figure 19 Molecular excitation spectra of cyclo-WW in different solvents in the absence or presence of Zn(II). By comparing cyclo-WW in MeOH (black), cyclo-WW+Zn(II) in MeOH (blue) and in DMSO (red), it was found that the molecular excitation red-shifted from 305 nm in MeOH to 310 nm in DMSO (emission set at 350 nm), demonstrating that cyclo-WW+Zn(II) self-assembled more extensively in DMSO than in MeOH.

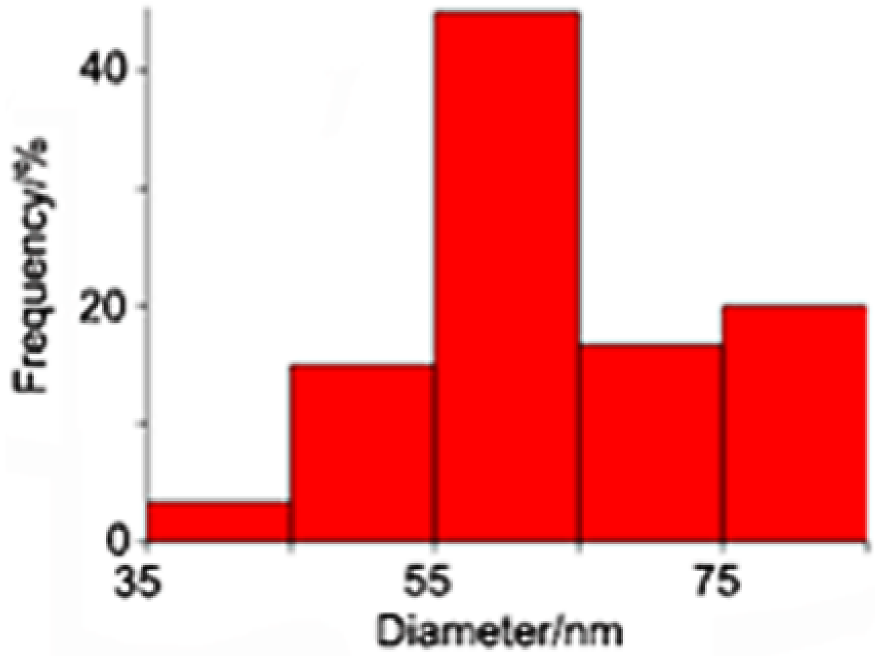

Supplementary Figure 20 Statistical diameter distribution of cyclo-WW+Zn(II) nanospheres in DMSO, as analyzed from TEM images. The results showed a diameter of 63.6 ± 12.2 nm.

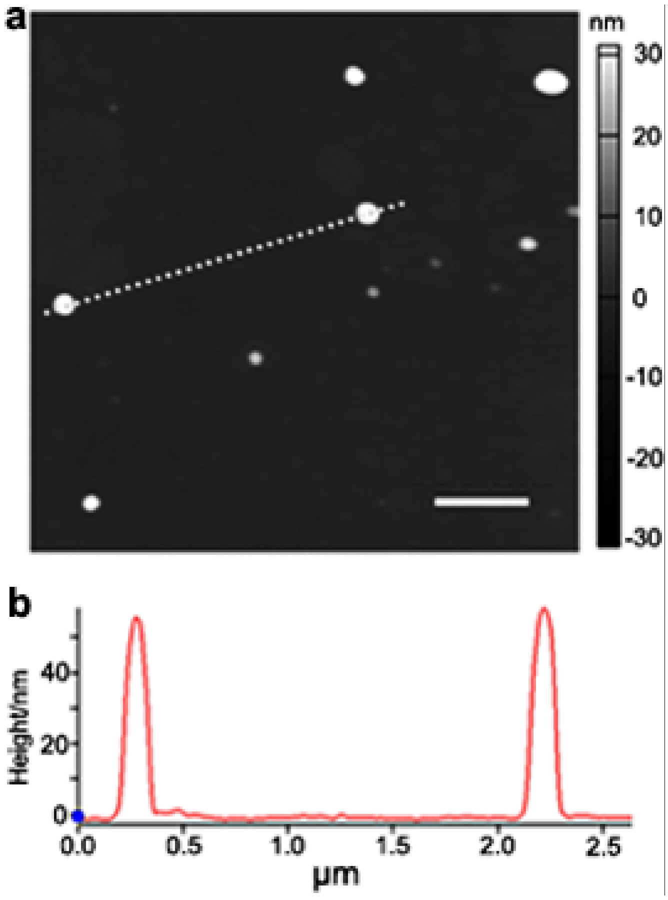

Supplementary Figure 21 AFM characterization of cyclo-WW+Zn(II) self-assemblies in DMSO. a, AFM image of the nanospheres. Scale bar: 500 nm. **b,** Cross-section profile corresponding to the dotted white line in the AFM micrograph shown in (**a**), demonstrating that the nanospheres were approximately 60 nm in size, consistent with TEM results.

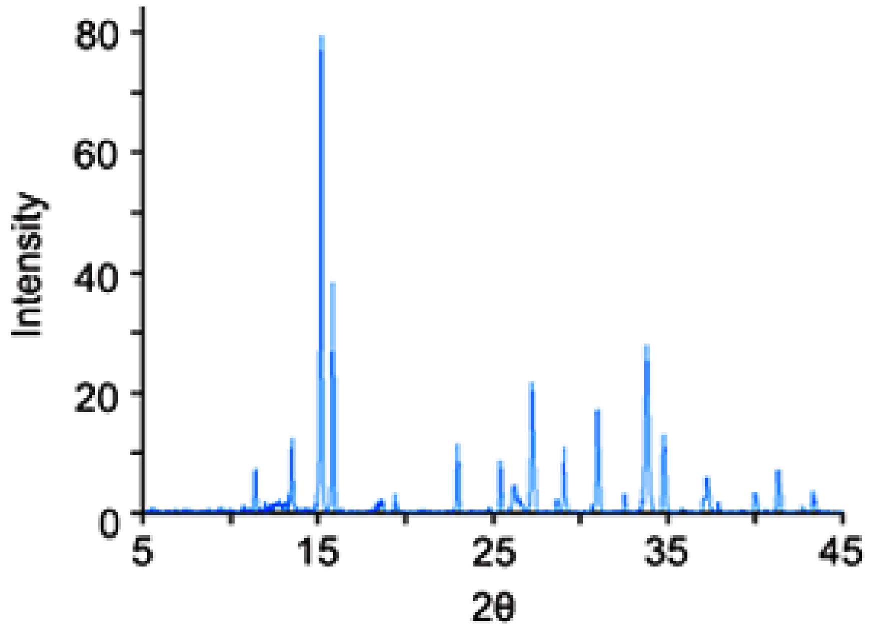

Supplementary Figure 22 Powder XRD spectrum of the nanospheres formed by cyclo-WW+Zn(II) in DMSO. The distinct sharp peaks and high intensities indicate well-ordered nanocrystal structures within the self-assemblies.

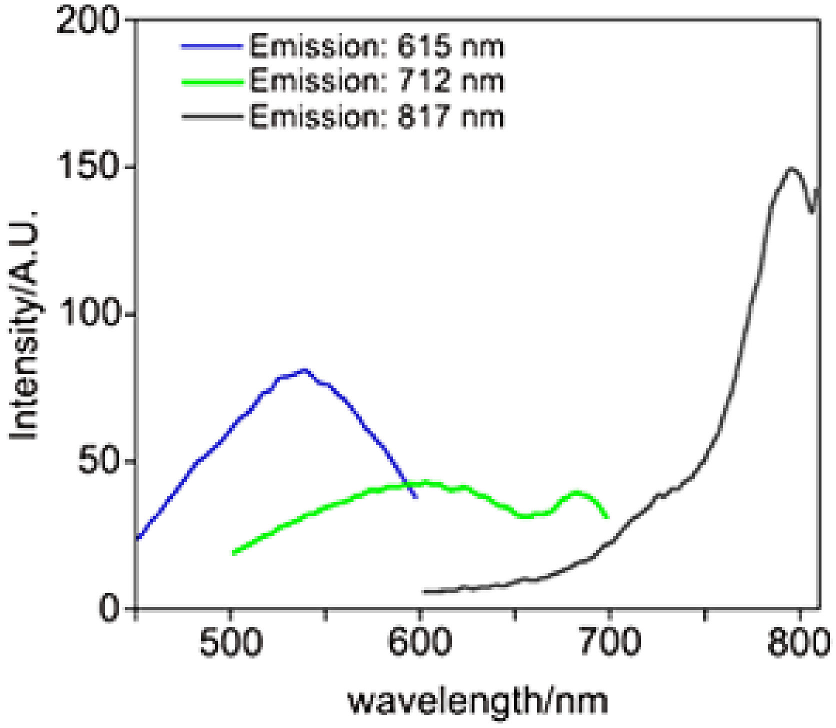

Supplementary Figure 23 Fluorescent excitation spectra of cyclo-WW+Zn(II) in DMSO at various emission wavelengths, as indicated.

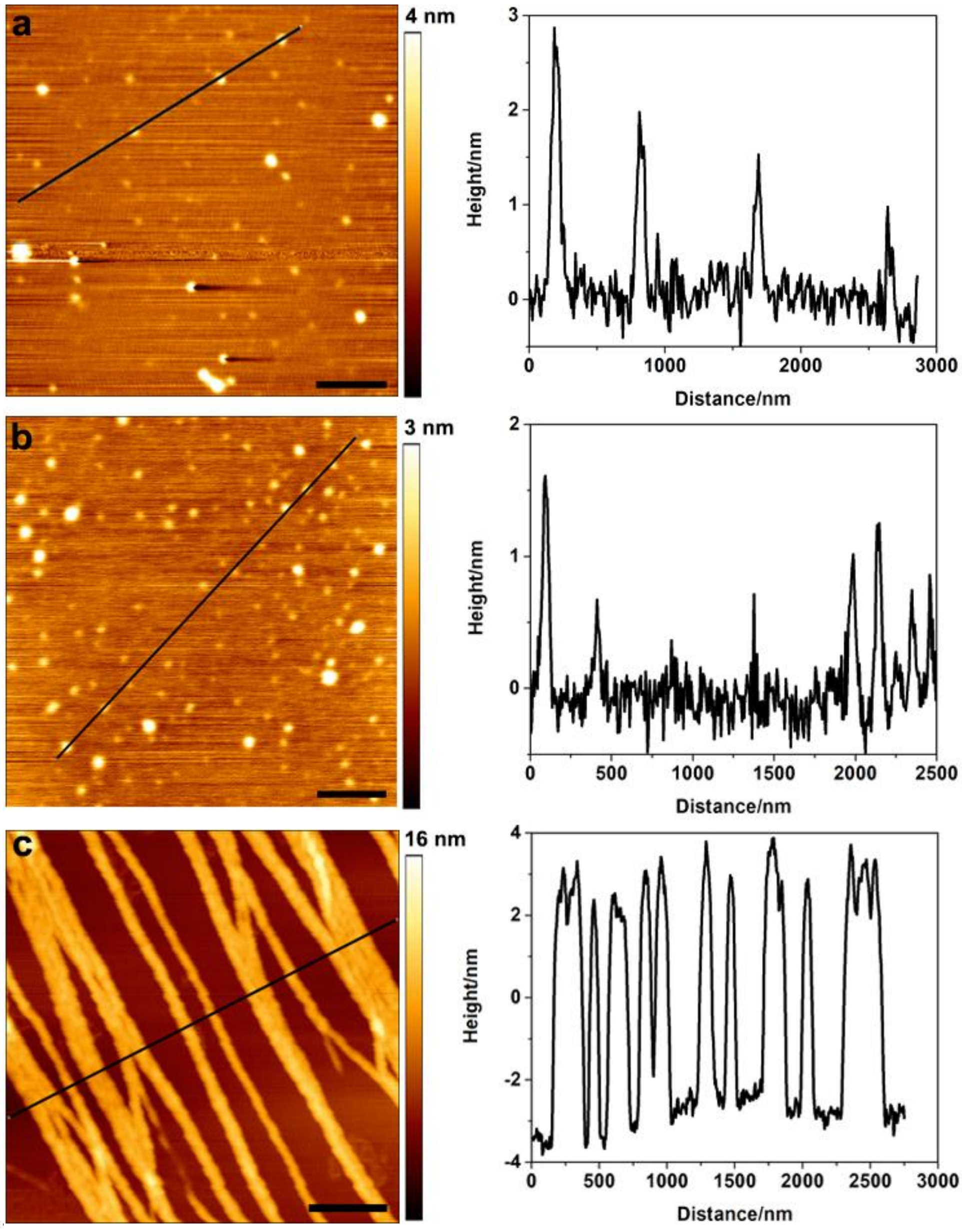

Supplementary Figure 24 AFM images showing the morphologies self-assembled by aromatic cyclo-dipeptides at 0.5 mM. a, cyclo-HH in DMSO. Scale bar: 600 nm. **b,** cyclo-FF in DMSO. Scale bar: 400 nm. **c,** cyclo-YY in DMSO. Scale bar: 500 nm. The cross-section profiles are corresponding to the black lines in the AFM micrographs.
